# *Cryptosporidium* spp. Infection in Adult Kidney Transplant Patients: A Systematic Review and Meta-Analysis

**DOI:** 10.3390/jcm13216395

**Published:** 2024-10-25

**Authors:** Danuta Kosik-Bogacka, Natalia Łanocha-Arendarczyk, Krzysztof Korzeniewski, Maciej Mularczyk, Joanna Kabat-Koperska, Paweł Ziętek, Małgorzata Marchelek-Myśliwiec

**Affiliations:** 1 Independent Laboratory of Pharmaceutical Botany, Pomeranian Medical University in Szczecin, Powstańców Wielkopolskich 72, 70-111 Szczecin, Poland; 2 Department of Biology and Medical Parasitology, Pomeranian Medical University in Szczecin, Powstańców Wielkopolskich 72, 70-111 Szczecin, Poland; natalia.lanocha.arendarczyk@pum.edu.pl; 3 Department of Epidemiology and Tropical Medicine, Military Institute of Medicine, 04-141 Warsaw, Poland; kkorzeniewski@wim.mil.pl; 4 Department of Gross Anatomy, Pomeranian Medical University in Szczecin, Powstańców Wielkopolskich 72, 70-111 Szczecin, Poland; maciej.mularczyk@pum.edu.pl; 5 Clinic of Nephrology, Transplantology and Internal Medicine, Pomeranian Medical University in Szczecin, 70-111 Szczecin, Poland; joanna.kabat.koperska@pum.edu.pl (J.K.-K.); malgorzata.marchelek@gmail.com (M.M.-M.); 6 Department of Orthopaedics, Traumatology and Orthopaedic Oncology, Pomeranian Medical University in Szczecin, Unii Lubelskiej 1, 71-252 Szczecin, Poland; pawel.zietek@pum.edu.pl

**Keywords:** adult, *Cryptosporidium* spp., diarrhea, kidney transplantation

## Abstract

**Background:** Diarrhea frequently occurs after vascular organ transplantation, including kidney transplants. This may result from non-infectious factors, adverse effects of immunosuppressive medications, or infections caused by various pathogens, including viruses, bacteria, fungi, or parasites, for example, intestinal protozoan parasites such as *Cryptosporidium* spp., which are particularly dangerous for immunocompromised patients. **Methods:** This review is based on scientific articles sourced from validated databases such as PubMed, the National Center for Biotechnology Information (NCBI), ScienceDirect, and Google Scholar. The primary search was conducted on 12–13 July 2024, using the keywords ‘*Cryptosporidium*’ AND ‘cryptosporidiosis’ AND ‘kidney’ AND ‘transplant’ AND ‘adult’. Inclusion criteria encompassed human studies, case reports, peer-reviewed journal publications, review articles, and research articles in English. Exclusion criteria included studies not in English, gray literature (e.g., conference proceedings and abstracts), and data related to pediatric patients (under 18 years old) and HIV patients. **Results:** This systematic review and meta-analysis have highlighted an often-overlooked connection between *Cryptosporidium* spp. infections in adult kidney transplant recipients (KTR). Furthermore, it includes an analysis of the clinical presentation, diagnosis, and treatment of *Cryptosporidium* spp. infection in these patients, based on available case reports. Our study demonstrates that adult kidney transplant patients are at a significantly higher risk of acquiring *Cryptosporidium* spp. compared to healthy participants. **Conclusions:** *Cryptosporidium* spp. infections can be asymptomatic, making it essential to screen both symptomatic and asymptomatic kidney transplant recipients. The clinical presentation of cryptosporidiosis typically involves digestive symptoms and can be complicated by biliary tract involvement. In KTR patients presenting with diarrhea, it is crucial to not only test for *Cryptosporidium* spp. but also to rule out bacterial and viral etiologies, including infections such as *C. difficile*, *C. colitis*, *Clostridium* spp., and rotavirus. The diagnosis of *Cryptosporidium* spp. infections primarily relies on microscopic methods, which are known for their low sensitivity. Therefore, diagnostic approaches should include both direct methods and, where possible, molecular techniques. Based on the analyzed cases, the most effective treatment results were achieved with reduction in immunosuppression if possible (strong, very low) and nitazoxanide at a dose of 500 mg twice daily for 14 days. Considering the public health implications of our findings, the current epidemiological data underscore the need for further research to develop effective prevention and intervention strategies against cryptosporidiosis. Preventive measures, regular screening programs, and the treatment of *Cryptosporidium* spp. infections should be integrated into the clinical care of transplant patients. It is also important that patients are informed about environmental risk factors.

## 1. Introduction

In recent years, the survival rates of kidney transplant recipients (KTRs) have improved significantly, largely due to advances in preventing acute rejection through immunosuppressive therapy. However, while effective, immunosuppressive treatment can lead to immune suppression, increasing the risk of opportunistic infections, including those caused by bacteria, viruses, fungi, and parasites. Among the protozoan pathogens, *Entamoeba histolytica*, *Giardia duodenalis*, *Cyclospora* spp., *Cystoisospora* spp., *Cryptosporidium* spp., and microsporidia such as *Enterocytozoon bieneusi* are well-known causes of diarrhea and represent a significant public health concern, particularly in developing countries [1]. Notably, *Cryptosporidium* spp. is recognized as the second leading cause of diarrhea worldwide, following rotavirus infection [2].

The coccidian protozoan *Cryptosporidium* spp. is an opportunistic intestinal parasite that infects a wide range of vertebrate animals. Infections have been documented in amphibians, reptiles, poultry, and mammals, including humans [3]. To date, more than 40 species of *Cryptosporidium* with over 60 valid genotypes have been identified [4]. The Centers for Disease Control and Prevention (CDC) recognize *Cryptosporidium* spp. as an emerging protozoan parasite [5]. The majority of human infections are caused by *Cryptosporidium parvum*, which infects both humans and ruminants, and *C. hominis*, which primarily infects humans and pigs [6]. However, cases of cryptosporidiosis caused by other species and genotypes, such as *C. meleagridis*, *C. felis*, *C. canis*, *C. cuniculus*, *C. ubiquitum*, *C. viatorum*, *C. muris*, *C. suis*, *C. fayeri*, *C. andersoni*, *C. bovis*, *C. scrofarum*, *C. tyzzeri*, *C. erinacei*, and the *Cryptosporidium* horse, skunk, and chipmunk I genotypes have also been reported [7].

Cryptosporidiosis has been documented in over 40 countries across six continents, affecting both immunocompetent and immunocompromised individuals [8]. The infection is particularly prevalent among immunosuppressed patients, including those with HIV/AIDS, individuals undergoing chemotherapy, and solid organ transplant (SOT) recipients. Globally, the prevalence of cryptosporidiosis is estimated at 7.6% [7]. The frequency of *Cryptosporidium* spp. infection varies depending on factors such as study design, geographic location, population group, and the sensitivity and specificity of laboratory methods [9]. The prevalence is reported to be 1–3% in Europe and North America and 5–10% in Asia and Africa [10]. Cryptosporidiosis typically presents as an acute, self-limiting gastrointestinal disease characterized by abdominal pain, diarrhea, vomiting, low-grade fever, and anorexia. However, in immunocompromised individuals, the infection can persist, leading to severe, potentially fatal outcomes. Consequently, cryptosporidiosis is regarded as one of the most hazardous opportunistic infections in patients with acquired immunodeficiency syndrome [11]. Cryptosporidiosis primarily affects the gastrointestinal tract, with respiratory cryptosporidiosis occurring much less frequently. In immunosuppressed patients, the severity of the infection can be influenced by host factors such as age and nutritional status, as well as the specific species and subtype of *Cryptosporidium* spp. involved [12]. Studies have shown that a CD4+ T-cell count of less than 200/mm^3^ increases the risk of prolonged infection, while counts below 100/mm^3^ may lead to severe, life-threatening diarrhea [13]. Respiratory tract infections caused by *Cryptosporidium* spp. have been documented in immunodeficient patients.

The *Cryptosporidium* spp. exhibit a monoxenous life cycle, meaning they complete their entire life cycle within a single host. Both asexual and sexual stages occur in the intestinal epithelium, leading to the production of two forms of oocysts. Thin-walled oocysts are responsible for autoinfection, while thick-walled oocysts are excreted in the feces, facilitating transmission to new hosts [14]. Oocysts containing sporozoites are shed in the feces of infected hosts. The infectious dose is estimated to be as low as 10–30 oocysts [15], though some researchers suggest that even a single oocyst can cause infection, particularly in immunocompromised individuals [16].

*Cryptosporidium* oocysts can be transmitted through various routes, including direct or indirect human-to-human or animal-to-human contact, and through contaminated water and food. Transmission may occur via ingestion through animal contact, consumption of contaminated water or recreational water, travel to disease-endemic regions, poor hygiene, or foodborne routes [16]. Airborne transmission and mechanical transport of *Cryptosporidium* spp. oocysts by flies and other insects have also been explored [11,17,18]. The environmental dissemination of *Cryptosporidium* spp. oocysts is facilitated by the large number of oocysts shed by the host. These oocysts are highly resistant to environmental factors and standard disinfection methods [19]. Once ingested, the oocysts excyst in the small intestine, releasing sporozoites that invade enterocytes.

**Figure 1:**
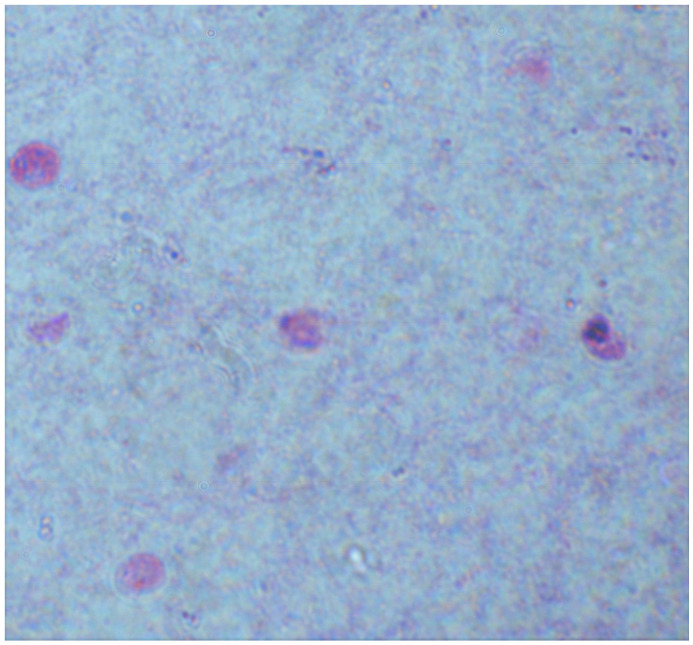
Oocysts of *Cryptosporidium* spp. in a direct smear (modified Ziehl–Neelsen staining, ×1000).

For the diagnosis of *Cryptosporidium* spp., stool samples can be used in various forms, including fresh, frozen, and formalin-fixed. However, stool samples fixed with polyvinyl alcohol (PVA) are not suitable for some staining techniques and are generally not used for molecular diagnosis. Most laboratories rely on microscopic methods for diagnosing cryptosporidiosis, which typically involve detecting the presence of *Cryptosporidium* spp. oocysts in samples using light microscopy (Figure 1) or phase-contrast microscopy [20].

The sensitivity of microscopic methods for detecting *Cryptosporidium* spp. can be significantly enhanced by concentrating oocysts in stool samples through techniques such as centrifugation (1200× *g*), Sheather’s sugar flotation method, saturated salt flotation, and the Allen and Ridley formalin-ether method [20]. However, despite the use of these concentration methods, the effectiveness of the diagnosis largely depends on the expertise of the person evaluating the sample. Additionally, these methods may still fall short in detecting *Cryptosporidium* spp. oocysts in cases of asymptomatic infections, where the oocyst count in stool samples may be too low for reliable detection. Immunofluorescence antibody (IFA) staining techniques, which use monoclonal antibodies against the oocyst wall antigen, are also employed for the diagnosis of *Cryptosporidium* spp. These techniques are characterized by high sensitivity and are often more cost-effective compared to traditional staining methods [21].

Serological methods, which detect *Cryptosporidium* antigens or antibodies directed against this pathogen, offer even higher sensitivity and specificity than microscopic techniques [22]. Antigen detection can be performed using fluorescently or enzymatically labeled antibodies. These serological methods are particularly advantageous for screening large numbers of samples, making them invaluable in epidemiological studies. 

Coproantigen detection kits, which test for *Cryptosporidium* spp. antigens alone or in combination with *Giardia intestinalis* and/or *Entamoeba histolytica*, are commonly used in clinical settings [23]. Additionally, the detection of antibodies against *Cryptosporidium* spp. specific antigens in serum, saliva, or stool samples provides an indirect diagnostic method. The detection of specific antibodies is most useful in cases of seroconversion, where there is an observable increase in antibody titer or a change in antibody isotype, indicating a recent or ongoing infection.

Polymerase chain reaction (PCR), quantitative Real-Time PCR (qRT-PCR), restriction fragment length polymorphism (PCR-RFLP), multiplex allele-specific-PCR (MAS-PCR), and quantitative real-time PCR are advanced molecular techniques commonly used for the detection and characterization of various *Cryptosporidium* spp. genetic markers, including 18S rRNA, TRAP C1, COWP, Hsp 70, DHFR, and the glycoprotein (GP) 60 gene, as well as minisatellite and microsatellite markers, which are employed for species identification and the analysis of extrachromosomal double-stranded RNA elements [20].

Due to the robust structure of the oocyst wall, the DNA isolation procedure for *Cryptosporidium* spp. requires additional steps to effectively break down the wall. These steps may include initial homogenization, mechanical homogenization using glass beads, enzymatic lysis, alternating cycles of freezing and thawing of biological material, or incubation at temperatures above 70 °C [21].

For species determination, the analysis of the rRNA small subunit locus by PCR-RFLP is commonly used. Subtypes within species can be identified using PCR-RFLP or sequencing of polymorphic loci, with the Cpgp40/15 locus, also known as GP60, being the most frequently targeted [24].

Fluorescent in situ hybridization (FISH) is another method that can be applied for the detection of *Cryptosporidium* spp. This technique uses oligonucleotide probes to detect the presence of 18S rRNA sequences. However, FISH is generally more time consuming and less sensitive compared to other molecular methods, making it less commonly used in routine diagnostics [25].

Histopathological examination, utilizing autopsy and biopsy materials, can also be employed to identify the parasite [26]. While special stains are not required for tissue sections, they can facilitate the screening process [20].

**Figure 2:**
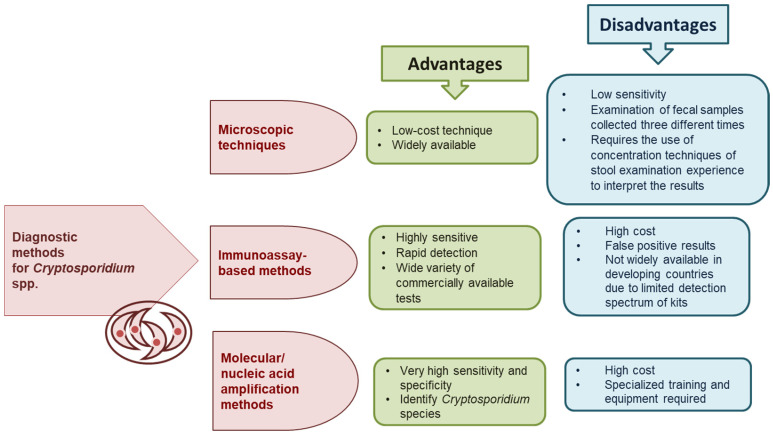
Advantages and disadvantages of diagnostic methods for *Cryptosporidium* spp. [22,27,28,29].

The advantages and disadvantages of microscopic, immunological, and molecular diagnostic methods for *Cryptosporidium* spp. are shown in Figure 2.

**Figure 3:**
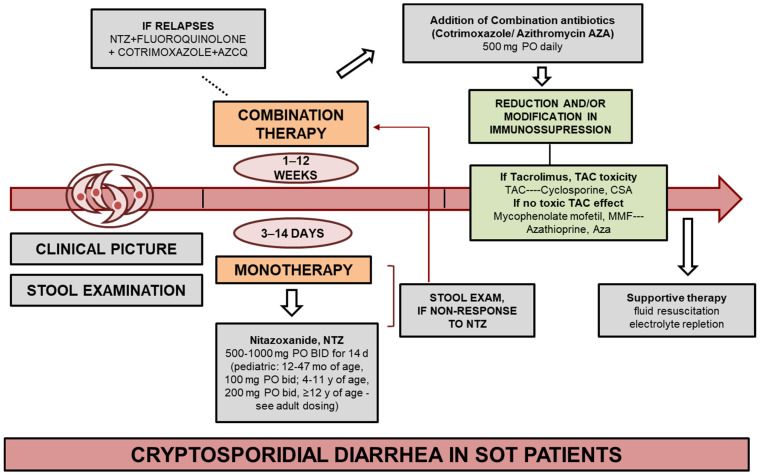
Treatment algorithm for *Cryptosporidium* spp. infection in patients after solid organ transplantation (SOT).

Treatment options for cryptosporidiosis, including in transplant recipients, are limited, which may be partly due to the lack of certain organelles in *Cryptosporidium* spp., such as the apicoplast, which is the target of many pharmacological therapies [30]. Various drugs are employed in the treatment of cryptosporidiosis, including nitazoxanide (NTZ), paromomycin, trimethoprim/sulfamethoxazole, rifampin, and fluoroquinolones, either used individually or in combination. However, the effectiveness of these treatments can vary, with patients experiencing different response durations [31] (Figure 3).

Nitazoxanide (NTZ) is the only drug approved by the Food and Drug Administration (FDA) for the treatment of diarrhea caused by *Cryptosporidium* spp. [32]. The drug primarily works by inhibiting pyruvate ferredoxin oxidoreductase, an enzyme crucial for electron transport in anaerobic energy metabolism. However, this may not be the sole mechanism of its action. Randomized studies have shown that NTZ can lead to a quicker resolution of symptoms and reduction in oocyst excretion [33,34]. NTZ is available in 500 mg tablets or as a 100 mg/5 mL suspension, and its bioavailability is increased when taken with food.

## 2. Cryptosporidiosis in Adult Kidney Transplant Patients

Few studies have focused on the occurrence of *Cryptosporidium* spp. in adult kidney transplant patients. Therefore, this study aimed to conduct a comprehensive meta-analysis and systematic review to assess the global status of *Cryptosporidium* spp. infection in this patient population. Additionally, an analysis of the clinical presentation, diagnosis, and treatment of *Cryptosporidium* spp. infection in adult kidney transplant recipients was performed based on case reports.

### 2.1. Methods

This review is based on scientific articles sourced from validated databases such as PubMed, the National Center for Biotechnology Information (NCBI), ScienceDirect, and Google Scholar. The primary search was conducted on 12–13 July 2024, using the keywords ‘*Cryptosporidium*’ AND ‘cryptosporidiosis’ AND ‘kidney’ AND ‘transplant’ AND ‘adult’. Inclusion criteria encompassed human studies, case reports, peer-reviewed journal publications, review articles, and research articles in English. Exclusion criteria included studies not in English, gray literature (e.g., conference proceedings and abstracts), and data related to pediatric patients (under 18 years old) and HIV patients. The literature search adhered to the PRISMA guidelines [35].

For each study included in the meta-analysis, odds ratios and 95% confidence intervals were calculated. The analysis was based on qualitative data, and a random-effects model was applied to assess the parameters of the meta-analysis. Results were presented in a forest plot. Heterogeneity between studies was determined using I^2^ and Cochran’s Q test. Results with *p*-values less than 0.05 were considered statistically significant.

For the statistical analysis of the prevalence of *Cryptosporidium* spp., patient age, time to onset of diarrhea post-kidney transplantation, and the duration of treatment, the STATISTICA software package (StatSoft Inc., Tulsa, OK, USA, version 10.0) was utilized. Qualitative variables were presented as counts and percentages.

### 2.2. Results and Discussion

**Figure 4:**
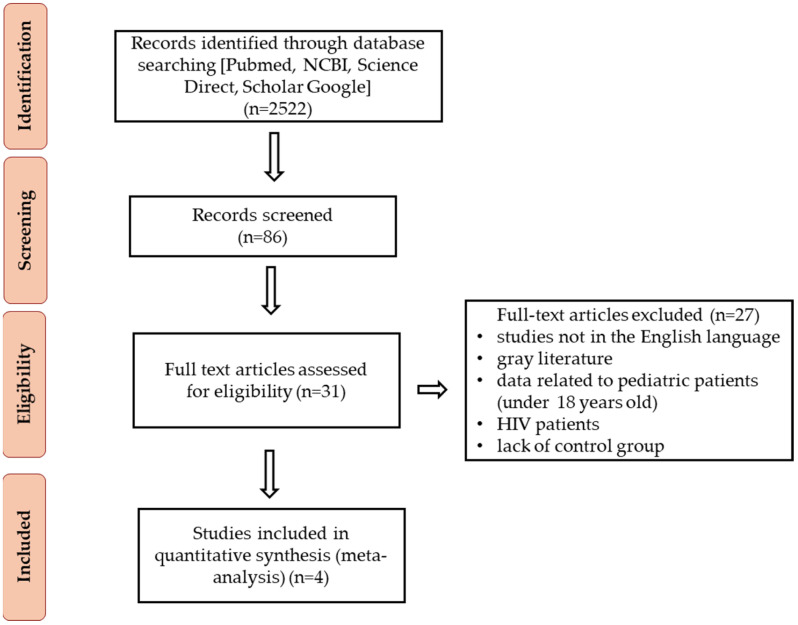
Flow diagram of literature search and selection.

A total of 2522 abstracts were reviewed, and 16 full-text articles were evaluated for inclusion in the meta-analysis (*n* = 3) and systematic review (*n* = 12), and one paper was included in both a meta-analysis and a systematic review. The articles were assessed for eligibility, resulting in 87 records after the full-text review. We then excluded 65 records due to insufficient statistical information and because they included data on pediatric patients or a mix of pediatric and adult patients. Our final dataset comprised 22 studies published between 1997 and 2023. The analyzed studies were divided into two groups: (1) epidemiological studies (meta-analysis) (Figure 4) and (2) clinical case reports.

#### 2.2.1. Epidemiological Research

The prevalence of human cryptosporidiosis varies across different populations. The infection rate of *Cryptosporidium* spp. in immunocompetent individuals in developing countries is higher (4–20%) than in developed countries (0.6–20%). Similarly, in immunosuppressed individuals, the prevalence ranges from 1.3 to 31.5% in developing countries compared to 0.1–14.1% in developed countries [36]. The National Reference Center in France reported that between 2017 and 2019, 40% of cryptosporidiosis cases with documented immune status occurred in immunodeficient patients, including 53% in SOT recipients. The majority of data regarding the prevalence of cryptosporidiosis in organ transplant recipients pertain to renal transplant patients. Based on epidemiological data from nine available scientific publications that met the inclusion criteria, the prevalence of cryptosporidiosis was found to be higher in kidney transplant recipients (6.25%) compared to immunocompetent individuals (1.45%) [37,38,39,40,41,42,43,44,45].

**Figure 5:**
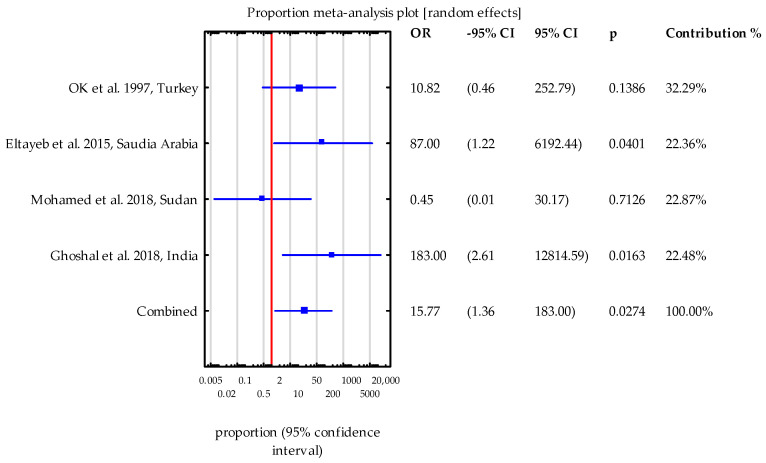
Forest plot diagram: the estimated pooled prevalence of *Cryptosporidium* spp. infection in adult kidney transplant patients, derived from a random-effects meta-analysis of the included studies, is shown. The studies included are based on staining methods [37,38,41,42] and the PCR technique [38]. The diagram displays data organized by the first author, year of publication, and country.

Four studies were selected for meta-analysis, which included results from both test and control groups. Heterogeneity between the studies was assessed using the I^2^ statistic (38.08%) and Cochran’s Q test (Q = 4.69; *p* = 0.195), indicating that the individual studies did not differ significantly from one another. In three of the four studies (with the exception of Mohamed et al. [41]), the number of patients infected with *Cryptosporidium* spp. who exhibited symptoms of diarrhea was higher compared to the control group. In two of these studies, specifically Eltayeb et al. [37] and Ghoshal et al. [38], the odds ratios (ORs) were notably high and statistically significant, with a wide range of 95% confidence intervals (OR = 87.00 and 183.00, respectively). The combined OR was statistically significant (OR = 15.77), indicating a substantially higher likelihood of diarrhea symptoms in patients infected with *Cryptosporidium* spp. A flowchart illustrating the study design process is provided (Figure 5).

#### 2.2.2. Analysis of Case Reports

Due to the lack of extensive clinical data on the course and treatment of kidney transplant recipients infected with *Cryptosporidium* spp. in epidemiological studies, the focus was shifted to case reports. From 1997 to 2023, 28 symptomatic cases of kidney transplant recipients with cryptosporidiosis caused by *Cryptosporidium* spp. were documented. Thirteen articles were selected for analysis (Table 1). The majority of patients originated from the USA and France, with additional cases reported from India, Spain, Turkey, Italy, Saudi Arabia, and Singapore (Table 1).

The cases described included 20 men and 8 women, with an average patient age of approximately 47 years (Table 2).

Different therapeutic strategies used to maintain immunosuppression after organ transplantation include corticosteroids, antibodies, calcineurin inhibitors (CNIs), anti-metabolite agents, and mammalian target of rapamycin (mTOR) inhibitors. The choice of these therapies depends on the protocols followed by various transplant centers worldwide. Achieving the optimal level of immunosuppression in SOT requires a delicate balance between preventing rejection and managing the side effects of immunosuppression [58,59]. The type of immunosuppressants used in kidney transplant patients may influence the course of cryptosporidiosis. It has been shown that patients using a tacrolimus-based regimen are at greater risk of *Cryptosporidium* spp. infection compared with a cyclosporine-based regimen [60]. In patients receiving tacrolimus with confirmed *Cryptosporidium* spp. infection, renal graft dysfunction is more common, possibly due to dehydration and increased tacrolimus concentration [57,61]. In patients receiving tacrolimus with diarrhea, blood levels of this drug should be assessed [62].

KRT patients with cryptosporidiosis more often received triple immunosuppressive drugs than those treated with double or single immunosuppressants [63]. The kidney transplant patients in the analyzed papers were most commonly treated with TAC + MMF + PRED (*n* = 14), less frequently Anti-IL2R CMI + MMF (*n* = 4), CNI + MMF (*n* = 2), TAC + MMF (*n* = 1), CYA + AZA + PRED (*n* = 1), TAC + SLM + PRED (*n* = 1), TAC + PRED + AZA (*n* = 1), and PRED + CNIs + MMF (*n* = 1) (Table 1). For three patients, no data were available regarding post-transplant immunosuppressive therapy. But in the study by Mohamed et al. [41], *Cryptosporidium* infection was found in KTR patients who were treated with CYA + AZA and CYA + Cortisone (CORT) + MMF. By contrast, no *Cryptosporidium* infection was found in patients treated with TAC + MMF and CYA + CORT.

Diarrhea is common after kidney transplantation, occurring in 10–50% of patients [64]. It has been observed that diarrhea associated with immunosuppressive therapy is more prevalent in the early post-transplant period due to the administration of multiple drugs, often in higher doses. Conversely, infectious diarrhea tends to manifest several years after transplantation [65].

*Cryptosporidium* infection can be asymptomatic and self-limiting, which may contribute to its low detectability. It was found that in KTR patients prevalence symptomatic *Cryptosporidium* infection was higher compared to the control group [42]. In cases of asymptomatic infection, *Cryptosporidium* spp. was identified in the distal parts of the small intestine and proximal parts of the colon [66]. Symptoms of cryptosporidiosis typically appear 1–14 days post-infection (average of 7 days) and persist for up to 6–9 days [67]. However, in some cases, symptoms can last for up to 100–120 days [68]. Patients infected with *Cryptosporidium* spp. usually excrete oocysts in their feces for an average of 7 days (ranging from 1 to 15 days) after the symptoms subside, with rare cases of oocyst excretion continuing for up to 2 months [11].

The most common symptom of cryptosporidiosis is severe watery diarrhea, which can occur up to 10 times a day and may be accompanied by mucus. Immunocompetent patients typically experience a self-limiting illness, whereas immunosuppressed patients, especially those with T-cell deficiencies, often develop chronic and severe cryptosporidiosis, with a risk of the disease spreading beyond the intestines [50]. The pathophysiology of diarrhea in *Cryptosporidium* spp. infection is not fully understood, but it is thought to involve a combination of malabsorption and secretory diarrhea, resulting from factors such as mucosal attachment, distortion of villous architecture, epicellular infection, inflammatory responses, and cellular apoptosis [69].

In renal transplant patients, post-transplant cryptosporidiosis with diarrhea is a common complication [70]. However, some researchers argue that in renal transplant recipients, cryptosporidiosis does not typically present with unusually severe symptoms or involve extraintestinal sites [71,72].

Watery diarrhea was observed in the majority of analyzed kidney transplant recipients (*n* = 27), with an average of 4–10 stools per day (Table 1). This symptom typically appeared approximately 28 months after kidney transplantation, with a median onset of around 31 months in men and 21 months in women, suggesting that patients were likely infected but asymptomatic prior to transplantation. Arlan et al. [73] noted that KTR patients with *Cryptosporidium* spp. observed diarrhea in 1–6 months (28.6%) and 6 months (71.4%) after transplantation. Other common symptoms of *Cryptosporidium* spp. infection in these patients included abdominal pain (*n* = 13), weight loss (*n* = 11) with an average loss of about 6 kg, and vomiting (*n* = 10). Cryptosporidiosis was complicated by renal failure in six patients, most likely secondary to dehydration and hypotension [46,47,52].

Recurrence of cryptosporidiosis symptoms in immunocompromised patients may occur even after treatment, often due to inadequate eradication of the pathogen, which can result from incorrect medication or improper dosage. This incomplete eradication is particularly problematic when *Cryptosporidium* spp. persists in the bile ducts, allowing the infection to remain in the latent stage. If cryptosporidiosis goes unrecognized in immunocompromised patients, it can lead to severe complications such as debilitating diarrhea, epithelial infection of the bile ducts, gastritis, pancreatitis, primary sclerosing cholangitis, bile duct inflammation or cancer, and cirrhosis [9].

Cryptosporidiosis-associated biliary tract inflammation is a particularly serious clinical complication in patients with AIDS. Symptoms of this condition include abdominal pain, fever, and jaundice. These patients often exhibit increased alkaline phosphatase activity in serum, along with significant anatomical damage to the biliary system [66]. Furthermore, *Cryptosporidium* spp. has been implicated in the development of malignant cancers in the gastrointestinal tract [74]. Among the patients included in this study was the first reported case of *Cryptosporidium*-induced sclerosing cholangitis in a renal transplant recipient who was not HIV-infected [54].

Recipients of transplanted organs may become infected with *Cryptosporidium* spp. in three different ways: (i) transfer through transplantation, (ii) de novo infection, or (iii) reactivation of latent infection due to immunosuppression [75]. In the group of patients described, infection with *Cryptosporidium* spp. most likely occurred de novo during travel (*n* = 7), through contact with animals (*n* = 4), contaminated water (*n* = 4), and food (*n* = 2) (Table 1).

To minimize the risk of *Cryptosporidium* infection in KTR patients, several preventive measures are recommended. These include regular handwashing, avoiding contact with young pets and farm animals (especially calves), avoiding exposure to infected individuals, and limiting travel to areas where the infection is prevalent. Additionally, protection against waterborne *Cryptosporidium* spp. infections is crucial. This can be achieved by boiling drinking water and using filters that trap particles approximately 1 µm in size. Pasteurization of milk for about 15 s at a temperature of around 70 °C is also recommended, as this process effectively destroys oocysts [46].

Optical microscopy is currently regarded as the gold standard for diagnosing cryptosporidiosis. However, relying on unstained preparations presents significant challenges due to the small size of oocysts (3–8 μm) and their resemblance to other structures, such as debris, yeast forms, and other protozoa. Acid-Fast staining, particularly the modified Ziehl–Neelsen (mZN) technique, is predominantly used in clinical laboratories [76]. In the studies we reviewed on the prevalence of *Cryptosporidium* spp. in KTR, all epidemiological investigations utilized staining methods, including the Kinyoun Modified Acid-Fast and mZN techniques. Furthermore, among the case reports analyzed, the most frequently employed diagnostic method was Modified Acid-Fast staining (*n* = 24), with the mZN technique used in 16 of these cases. These methods, while effective, are time-consuming and require skilled diagnosticians. Although these techniques boast a specificity of 100%, their sensitivity varies between 37% and 79.1% [22]. This limitation is partly due to the potential for yeast cells, fungal spores, and bacteria to be mistakenly identified as *Cryptosporidium* spp. oocysts, despite the clear contrast provided by the red-stained oocysts against the green background [77]. Additionally, because oocyst excretion by the host is irregular, it is recommended to conduct stool tests three times at intervals of 2–3 days [78]. A single stool examination may lead to a false-negative result. Consequently, while microscopy remains a fundamental diagnostic tool, it is often considered suboptimal.

In immunocompromised patients with unexplained diarrhea, fluorescent staining techniques are frequently used, as these patients are prone to recrudescence following periods of remission [79]. The sensitivity and specificity of fluorescent staining are reported to be low [80]. However, in the studies reviewed, this method was employed in only one instance [52].

Complementary to staining methods are enzyme-linked immunosorbent assays (ELISAs) and enzyme immunoassays (EIAs). These methods have been found to significantly improve sensitivity (66–100%) and specificity (100%) and have the advantage of being more automated compared to conventional staining methods [28]. In the analyzed case reports, an EIA was used for the diagnosis of *Cryptosporidium* spp. infection in five patients (Table 1).

Molecular methods, particularly PCR, are gaining popularity in routine diagnostics for detecting *Cryptosporidium* spp. However, many studies have shown that molecular methods are still only used in a minority of routine diagnostic laboratories in Europe and the USA [22]. Molecular methods are more sensitive, with a detection range from 1 to 10^6^ oocysts. The sensitivity and specificity of commercial molecular diagnostic tests for *Cryptosporidium* spp. are 53.1–100% and 100%, respectively, and they also allow for the detection of other intestinal parasites [22]. In the publications included in the meta-analysis, PCR was utilized in only one epidemiological study [38], while this method was used for the diagnosis of *Cryptosporidium* spp. infection in nine KTR patients (Table 1).

The analysis of genotypes, subtypes, and clonality of *Cryptosporidium* could be valuable in understanding and determining the prognosis and severity of infections [26]. The shift from microscopy to molecular methods, which offer faster diagnosis and greater sensitivity, can also be attributed to the declining microscopy skills among personnel in modern clinical laboratories [81]. It seems likely that molecular methods will eventually completely replace other diagnostic methods.

Histological examination of intestinal mucosal biopsies is rarely used for routine diagnosis due to uneven parasite distribution in the biopsy, which can lead to false-negative results, and it is an expensive and time-consuming technique [20]. In three of the analyzed patients, an intestinal mucosal biopsy was taken during colonoscopy, which confirmed the presence of *Cryptosporidium* spp. (Table 1).

In only two of the analyzed studies were the species of *Cryptosporidium* identified in KTR patients. Among the adult kidney transplant recipients analyzed, infections with *C. parvum* (*n* = 5), *C. felis* (*n* = 2), and *C. hominis* (*n* = 1) were identified [46,47]. In terms of symptoms and treatment, the specific *Cryptosporidium* species appears to have little impact. But, when comparing clinical parameters between patients infected with *C. hominis* and *C. parvum*, patients with *C. hominis* infection more often had nausea and/or vomiting [63]. But fever, abdominal pain, and the frequency and duration of diarrhea were comparable between patients with *C. hominis* and *C. parvum* infection.

However, species identification is crucial in epidemiological and scientific research, as it allows for the analysis of *Cryptosporidium* spp. transmission. In cases of *C. parvum* infection, patients reported waterborne transmission (*n* = 2), travel (*n* = 1), and contact with animals (*n* = 1). The patient infected with *C. hominis* reported travel abroad, while the two patients infected with *C. felis* reported contact with animals (Table 1).

According to the literature, post-transplant patients face a significant risk of infections due to immunosuppressive therapy, which is a common challenge in the management of KTR [58,82]. It has been noted that post-transplant infections may follow a predictable pattern with respect to timing after transplantation. Early infections (within the first month post-transplant) are often related to nosocomially acquired pathogens, surgical complications, and some donor-derived infections. Opportunistic pathogens tend to emerge later, typically within the subsequent five months, as the effects of immunosuppressive therapies become more pronounced [83].

Co-infection is a common feature of parasitic infections in transplantation, and invasive disease may be associated with viral or disseminated bacterial infections, including antibiotic-resistant pathogens such as MRSA, VRE, and resistant Gram-negative bacilli. KTR patients are particularly susceptible to opportunistic infections, such as *Pneumocystis jirovecii* and cytomegalovirus (CMV) infections, due to prolonged immunosuppression and increased vulnerability to various pathogens [84]. In recent years, there have also been reports of bacterial or fungal co-infections with SARS-CoV-2 in KTR patients [85]. For instance, Shrateh et al. [50] reported co-infection of SARS-CoV-2 and *Cryptosporidium* spp. in a 41-year-old kidney transplant recipient.

The occurrence of co-infections in organ transplant patients, including kidney transplant recipients, may be influenced by multiple factors. One significant factor is the reduction in cyclosporine-based immunosuppressive regimens and the increased use of newer drugs that lack the anti-parasitic effects of cyclosporine metabolites, potentially leading to higher rates of parasitic infections [58]. Additionally, the reactivation of dormant infections may occur as a result of immunosuppression, which in transplant recipients is induced by drugs necessary to initiate immunosuppression in the early phase, maintain it in the late phase, or treat organ rejection [59]. It is estimated that 70% of KTR patients will experience a co-infection episode within the first three years after transplantation [84].

Among protozoan intestinal parasites, *Cryptosporidium* spp., *Cyclospora cayetanensis*, *Cystoisospora belli*, *Giardia* spp., *Blastocystis* spp., *Entamoeba histolytica*, *Dientamoeba fragilis*, and *Balantidium coli* are the most frequently observed in KTR patients [58]. However, such co-infections are rare in developed countries due to higher levels of hygiene, socioeconomic status, and the use of broad-spectrum medications. Among the 17 patients analyzed, tests for viral and bacterial infections were conducted in addition to parasitological examinations. Co-infections were detected in four patients, including two with bacterial co-infections (*Staphylococcus epidermidis* and *Campylobacter jejuni*) and two with viral co-infections (SARS-CoV-2 and norovirus) (Table 1).

For SOT recipients, the American Society of Transplantation’s Infectious Diseases Community of Practice recommends the use of NTZ for the treatment of cryptosporidiosis. Although data on the use of NTZ in transplant recipients are limited and primarily derived from case reports, some researchers have observed that long-term NTZ therapy can be effective [33,34]. In immunocompetent patients, NTZ is typically administered for three days, but in patients with compromised immunity, including KTR, the duration of treatment may be extended to up to 14 days [86].

Additionally, some studies have indicated that macrolides, such as azithromycin (AZCQ) and spiramycin, exhibit activity against *Cryptosporidium* and have shown promising results in transplant recipients [87]. Adjusting immunosuppressive therapy and carefully monitoring immunosuppressant levels are crucial steps in both the management and prevention of cryptosporidiosis in these patients. For example, Tie et al. [87] reported that in a patient who had undergone liver transplantation, NTZ therapy combined with controlled CD4+ T-cell counts between 100 and 300/mm^3^ was highly effective against *Cryptosporidium* without causing immunorejection.

A higher frequency of *Cryptosporidium* spp. infection has been observed in individuals taking tacrolimus compared to those on cyclosporine [57,60,87]. Additionally, it has been found that MMF may exhibit activity against *Cryptosporidium* spp. by inhibiting folate metabolism [88]. When managing cryptosporidiosis in transplant recipients, consideration should be given to converting to a less potent immunosuppressive drug, such as cyclosporine, or reducing the doses of previously administered immunosuppressive medications if necessary [60].

Infection of the biliary tract in immunocompromised patients may act as an extraintestinal reservoir, contributing to a lack of response to certain treatments, such as paromomycin, and leading to potential relapses. For these patients, it is recommended to administer drugs that are excreted in bile, including NTZ, to enhance the effectiveness of treatment [89].

In the treatment of patients in the group described in this study, monotherapy with NTZ at a dose of 500 mg b.i.d. was most commonly used (*n* = 14). Additionally, monotherapy was employed with rifaximin (*n* = 1), paromomycin (*n* = 4), spiramycin (*n* = 2), and azithromycin (AZM) (*n* = 2). Combination therapies included AZM + NTZ (*n* = 2), NTZ + AZM + rifaximin, paromomycin + AZM, and AZM + NTZ + TMP-SMX in individual patients. Immunosuppressive therapy was reduced, modified, or discontinued in 20 patients (Table 1).

In the case of a patient with *Cryptosporidium*-induced sclerosing cholangitis following renal transplantation, treatment included ursodeoxycholic acid (UDCA) and reduction in immunosuppression, followed by the introduction of an antibiotic (rifampin, RIF), which resulted in the eradication of the parasite and improvement in both biochemical and clinical parameters (Table 1). The duration of treatment was documented for twelve patients, with an average treatment duration of 72 days (Table 2).

## 3. Limitations

The limitations of our study include its retrospective nature and the relatively small number of patients. Most studies in this area focus on children or include both children and adults. There is a lack of epidemiological research specifically addressing *Cryptosporidium* spp. infections in adult kidney transplant recipients, with most studies concentrating on patients with diarrhea. Additionally, the majority of studies in this field are conducted in developing countries, with a noticeable absence of data from developed countries. Case reports are only available from Europe and North America.

## 4. Conclusions

1. This systematic review and meta-analysis have highlighted an often-overlooked connection between *Cryptosporidium* spp. infections and kidney transplantation. Our study demonstrates that adult kidney transplant patients are at a significantly higher risk of acquiring *Cryptosporidium* spp. compared to healthy participants.
2. *Cryptosporidium* spp. infections can be asymptomatic, making it essential to screen both symptomatic and asymptomatic kidney transplant recipients. The clinical presentation of cryptosporidiosis typically involves digestive symptoms and can be complicated by biliary tract involvement.
3. In KTR patients presenting with diarrhea, it is crucial to not only test for *Cryptosporidium* spp. but also to rule out bacterial and viral etiologies, including infections such as *C. difficile*, *C. colitis*, *Clostridium* spp., and rotavirus.
4. The diagnosis of *Cryptosporidium* spp. infections primarily relies on microscopic methods, which are known for their low sensitivity. Therefore, diagnostic approaches should include both direct methods and, where possible, molecular techniques.
5. Based on the analyzed cases, the most effective treatment results were achieved with reduction in immunosuppression if possible (strong, very low) and nitazoxanide at a dose of 500 mg twice daily for 14 days.
6. Considering the public health implications of our findings, the current epidemiological data underscore the need for further research to develop effective prevention and intervention strategies against cryptosporidiosis. Preventive measures, regular screening programs, and the treatment of *Cryptosporidium* spp. infections should be integrated into the clinical care of transplant patients. It is also important that patients are informed about environmental risk factors.

## Figures and Tables

**Table 1:** Cases of cryptosporidiosis in adult kidney transplant patients as reported in the scientific literature (TAC: tacrolimus; MMF: mycophenolate mofetil; MPA: mycophenolic acid; PRED: prednisone; CNIs: calcineurin inhibitors; CYA: cyclosporine; RIX: rituximab; MAFST: Modified Acid-Fast Stain; SLM: sirolimus; mZN stain: Modified Ziehl–Neelsen stain; EIA: enzyme immunoassay; AZA: azathioprine; IL-2R: IL-2 receptor; CNI: calcineurin inhibitor; NTZ: nitazoxanide; AZM: azithromycin; MP-SMX: trimethoprim-sulfamethoxazole; RIF: rifampin; TMP-SMX: Co-Trimoxazole DS; UDCA: ursodeoxycholic acid; q.d.: daily; d: days; b.i.d.: twice daily; t.i.d.: three times daily; q.o.d.: every other day; d.d.: divided dose).

Country/ Year	Patient Age/Sex	Kidney Transplantation		*Cryptosporidium* spp. Infection					Treatment of Cryptosporidiosis		Reference
		Time After/Number of Grafts	Immunosuppressive Treatment	Diagnostic Methods	Species/Intensity	Coinfection	Symptoms	Risk Factor	Drugs	Time	
France, 2016	60/M	8 years/1st graft	TAC (4 mg/day) + MMF) (1 g × 2/day) + PRED (7.5 mg/day)	mZN stain, PCR	*C. felis*/5–10 oocysts/slide	No	watery diarrhea (2 weeks), nausea, vomiting, weight loss (6 kg)	contact with dog	NTZ 500 mg b.i.d. × 14 d	2 weeks	[46]
	64/M	2 years/1st graft	TAC (7 mg × 2/day) + MMF (750 mg × 2/day) + PRED (10 mg/day)	mZN stain, PCR	*C. hominis*/>100 oocysts/slide	ND	watery diarrhea, abdominal pain, weight loss (13 kg)	travel to Mali	1. Reduction in TAC	4 weeks	
									2. NTZ 500 b.i.d. × 14 d		
	34/M	10 days/2st graft	TAC (6 mg × 2/day) + MMF (750 mg × 2/day) + PRED (25 mg/day)	mZN stain, PCR	*C. parvum*/1–5 oocysts/slide	ND	watery diarrhea, abdominal pain weight loss (10 kg)	travel to Kosovo	1. Reduction in TAC	4 months	
									2. NTZ 500 mg b.i.d × 14 d		
France, 2014–2015	68/M	56 months/1st graft	Anti-IL2r CNI + MMF	mZN stain, PCR	*C. parvum*	No	diarrhea, vomiting, dehydration, weight loss (8 kg), acute kidney injury, acidosis	contact with animals and children	1. Reduction in MMF until diarrhea resolved (6 patients) 2. 500 mg NTZ b.i.d. × 4 weeks (3 patients)	2 weeks—3 patients, 4 weeks—3 patients	[47]
	42/F	25 months/1st graft	Anti-IL2r CNI + MMF	mZN stain and PCR	ND	No	fever, abdominal pain, diarrhea, vomiting, dehydration, weight loss (4 kg)	previous antibiotic therapy			
	77/M	14 days/1st graft	Anti-IL2r CNI + MMF		*C. parvum*	No	severe diarrhea, dehydration, weight loss (3 kg), acute kidney injury	contact with untreated water			
	53/M	2 days/1st graft	Anti-IL2r CNI + MMF		*C. felis*		diarrhea, vomiting, dehydration, weight loss (4 kg)	contact with cat			
	64/F	65 months/1st graft	Depleting therapy CNI + MMF		*C. parvum*	No	fever, abdominal pain, diarrhea, vomiting, dehydration, weight loss (2 kg)	none			
	37/F	57 months/3st graft	Desensitization, depleting therapy CNI + MMF		*C. parvum*	Norovirus	fever, abdominal pain, diarrhea, vomiting, dehydration, weight loss (3 kg)	work as a nurse, contact with recreative water, treated with phenoxymethylpenicillin/F			
Italy, 2005	42/F	1 year/1st graft	TAC (present levels 8–10 ng/mL) + MMF (1250 mg/day) + PRED (5 mg/day)	MAFST	No	ND	abdominal pain, diarrhea (1 week)	traveled to Cuba	Rifaximin 600 mg t.i.d.	1 year	[48]
Spain, 2017	57/M	11 months/1 st graft	PRED (5 mg daily) + CNIs + MMF	ND	ND	No	watery diarrhea (8–10 times per day), abdominal discomfort, and weight loss	ND	Paromomycin 700 mg t.i.d.	2 weeks	[49]
Turkey, 1997	38/M	2 years/1st graft	ND	MAFST	ND	ND	diarrhea (10 days)	ND	Spiramycin 2 g q.d. × 10 d	3 months	[42]
	42/M	1 year/1st graft	ND		ND	ND	abdominal pain, distention	ND	Spiramycin 2 g q.d. × 10 d	4 months	
Palestine, 2022	41/M	2 years/1st graft	ND	ND	ND	COVID-19	weakness, fever of 39 °C, yellowish diarrhea occurring 4–5 times daily without blood	ND	NTZ 500 mg mg b.i.d × 14 d	ND	[50]
Saudi Arabia, 2007	60/F	4 months/1st graft	TAC (2 mg b.l.d.) + PRED (10 mg q.d.) + TMP-SMX	Biopsis, PAS-stain	ND	No	watery diarrhea (6–7 times per day), colicky abdominal pain	ND	paramomycin 500 mg b.l.d × 1 months	1 months	[51]
Singapore, 2019	37/M	2 years/1st graft	PRED + MMF + TAC	MAFST	ND	No	acute diarrhea, up to 10 times daily (2 weeks), abdominal discomfort, coryzal symptoms	ND	paromomycin 1 g b.i.d. + AZM 500 mg q.d.	4 weeks	[52]
									2. MMF reduction to 1 g/day		
India, 2014	35/F	4 months	TAC 3 mg/d + MMF 2 g/d + PRED 10 mg	MAFST	ND	No	watery diarrhea	ND	1. NTZ 500 mg mg b.i.d. × 3 d, TAC reduction (2 mg/d)	4 weeks	[53]
							more than 2 weeks of profuse watery diarrhea, abdominal cramps, dehydration	ND	3. TAC reduction (1 mg/d), MMF reduction (1 g/d0, NTZ 500 mg mg b.i.d. and AZA 500 mg q.d		
								ND	3. NTZ 500 mg mg b.i.d. and AZA 500 mg q.d., MMF replaced AZA 100 mg		
	30/M	4 months	RIX	MAFST	ND	ND	diarrhea	ND	NTZ 500 mg mg d.d., AZA 500 mg q.d., TAC reduction (1.5 g/d)	4 weeks	
Canada, 2003	40/F	9 years/1st graft	TAC 3 mg b.i.d. + PRED 15 mg q.o.d. + AZA (50 mg d.)	ND	*Cryptosporidium*-induced sclerosing cholangitis	ND	diarrhea (4–6 watery stools per day), weight loss, marked itching, fatigue	ND	1. UDCA 15 mg/kg d., reduction in TAC to 2.5 mg b.i.d., AZA to 25 mg d.	3 months	[54]
									2. After 2 weeks, UDCA 15 mg/kg d, RIF 300 mg d × 3 weeks, after 2 weeks TAC increase to 10 mg b.i.d		
USA, 2005	59/F	2 weeks	TAC + SLM + PRED	Biopsies, *MAFST*	ND	ND	nausea, vomiting, cramps, abdominal pain, profuse diarrhea	ND	Paromomycin 1 g b.i.d. × 4 weeks, reduced immunosuppression	2 weeks	[55]
USA, 2020	24/M	1 month/2nd graft	TAC + MMF	Biopsies, EIA	ND	No	chronic watery diarrhea, fevers, chills, nausea	ND	1. NTZ 500 mg b.i.d × 7 d, exchange of MMF for AZA	10 days	[56]
									2. NTZ 1 g b.i.d. + AZM 600 mg d + rifaximin 550 mg b.i.d. + intravenous fluids + diphenoxylate-atropine		
USA, 2004–2010	51/M	48.3 months/1st graft	TAC + MMF + PRED	mZN stain	ND	No	diarrhea	travel	AZM 250 mg q.d. × 21 d	ND	[57]
	53/M	151 months/1st graft	CYA + AZA + PRED	EIA	ND	*Staphylococcus epidermidis*	diarrhea, malaise, vomiting	ND	None	ND	
	36/M	53.1 months	TAC + NNF + PRED	mZN stain	ND	No	diarrhea	travel	AZM 600 mg q.d. and NTZ 500 mg b.i.d. × 18 d; MMF was discontinued	ND	
	52/M	3.4 months	TAC + MMF + PRED	EIA	ND	No	diarrhea, malaise, abdominal pain, vomiting	restaurant	AZM 600 mg q.d. × 2 d and NTZ 500 mg b.i.d. × 6 d; MMF was discontinued	ND	
	36/M	34.8 months	TAC + MMF + PRED	EIA	ND		diarrhea, vomiting	well water, farm animals	AZM 250 mg q.d. × 14 d; MMF was discontinued	ND	
	57/M	22.1 months	TAC + MMF + PRED	mZN stain	ND	*Campylobacter jejuni*	diarrhea, malaise, abdominal pain, vomiting	travel	MMF dose reduction	ND	
	34/M	66.0 months	TAC + MMF + PRED	EIA	ND	ND	diarrhea, malaise	travel	AZM 600 mg q.d. × 5 d., NTZ 500 mg b.i.d. × 14 d., TMP-SMX × 14 d	ND	

**Table 2:** Characteristics of adult kidney transplant patients included in the analysis, based on the scientific literature [42,46,47,48,49,50,51,52,53,54,55,56,57].

Characteristics		Value (AM ± SD)
age of patients	total (*n* = 28)	47.25 ± 13.19
	male (*n* = 20)	47.20 ± 14.05
	female (*n* = 8)	47.38 ± 11.61
time to onset of diarrhea after kidney transplantation (in months)	total (*n* = 28)	28.69 ± 35.27
	male (*n* = 20)	31.64 ± 38.61
	female (*n* = 8)	21.30 ± 25.78
duration of treatment (days)	total (*n* = 12)	72.00 ± 101.03
	male (*n* = 9)	50.77 ± 47.02
	female (*n* = 3)	135.67 ± 198.73

## Data Availability

The data supporting the findings of this study are available from the corresponding author upon reasonable request.
